# The Carotid Endarterectomy Cadaveric Investigation for Cranial Nerve Injuries: Anatomical Study

**DOI:** 10.3390/brainsci11020211

**Published:** 2021-02-10

**Authors:** Orhun Mete Cevik, Murat Imre Usseli, Mert Babur, Cansu Unal, Murat Sakir Eksi, Mustafa Guduk, Talat Cem Ovalioglu, Mehmet Emin Aksoy, M. Necmettin Pamir, Baran Bozkurt

**Affiliations:** 1 Department of Neurosurgery, Acıbadem Mehmet Ali Aydinlar University, 34662 Istanbul, Turkey; omcevik@umn.edu (O.M.C.); murat.imre.usseli@acibadem.com (M.I.U.); murat.eksi@acibadem.com (M.S.E.); mustafa.guduk@acibadem.com (M.G.); necmettin.pamir@acibadem.edu.tr (M.N.P.); 2 Department of Neurosurgery, Bakırkoy Training and Research Hospital for Psychiatric and Nervous Diseases, Health Sciences University, 34147 Istanbul, Turkey; baburmert29@gmail.com (M.B.); talatcem@gmail.com (T.C.O.); 3 (CASE) Center of Advanced Simulation ant Education, Acıbadem Mehmet Ali Aydinlar University, 34684 Istanbul, Turkey; Cansu.Unal@live.acibadem.edu.tr (C.U.); Emin.Aksoy@acibadem.edu.tr (M.E.A.); 4 School of Medicine, Acıbadem Mehmet Ali Aydinlar University, 34684 Istanbul, Turkey

**Keywords:** carotid endarterectomy, cadaveric study, local venous anatomy, carotid endarterectomy postoperative morbidities, cranial nerve injuries

## Abstract

Cerebral stroke continues to be one of the leading causes of mortality and long-term morbidity; therefore, carotid endarterectomy (CEA) remains to be a popular treatment for both symptomatic and asymptomatic patients with carotid stenosis. Cranial nerve injuries remain one of the major contributor to the postoperative morbidities. Anatomical dissections were carried out on 44 sides of 22 cadaveric heads following the classical CEA procedure to investigate the variations of the local anatomy as a contributing factor to cranial nerve injuries. Concurrence of two variations was found to be important in hypoglossal nerve injury: the presence of a direct smaller vein in proximity of the carotid bifurcation, and the intersection of the hypoglossal nerve (HN) with this vein. Based on the sample investigated, this variation was observed significantly higher on the right side. Awareness of possible anatomical variations and early ligation of any small veins can significantly decrease iatrogenic injury risk.

## 1. Introduction

Cerebral stroke continues to be one of the leading causes of mortality and long-term morbidity [1]. Therefore, carotid endarterectomy (CEA) remains to be a popular treatment for both symptomatic and asymptomatic patients with carotid stenosis. The surgical technique regarding the plaque removal have long been debated and multiple pitfalls and thus complications have been listed for the procedure [2]. Although the results have improved over the last three decades, the incidence of cranial nerve injuries remain high due to the surgical area containing multiple vital structures and the wide variations that can be seen on these structures [3]. The most common of the injured nerves of the cranium remain to be hypoglossal, vagus and marginal mandibular branch of the facial nerve. The factors contributing to these injuries are local trauma created by retraction, stretching, clamping and transection. To lower the incidences, knowledge of the local anatomy should be at the utmost level. In the last 35 years, the incidence rate of cranial injuries has been lowered significantly, the hypoglossal nerve (HN) from 8% to 2% and the vagus from 8% to <1% [4]. Although the rates have been lowered significantly, they remain persistent, especially with the HN. In this study, the variations of the local anatomy as a contributing factor for postoperative cranial nerve injuries have been investigated. 22 formalin-fixed cadaveric heads were prepared and dissected following the steps of the classical CEA procedure.

## 2. Materials and Methods

**Figure 1:**
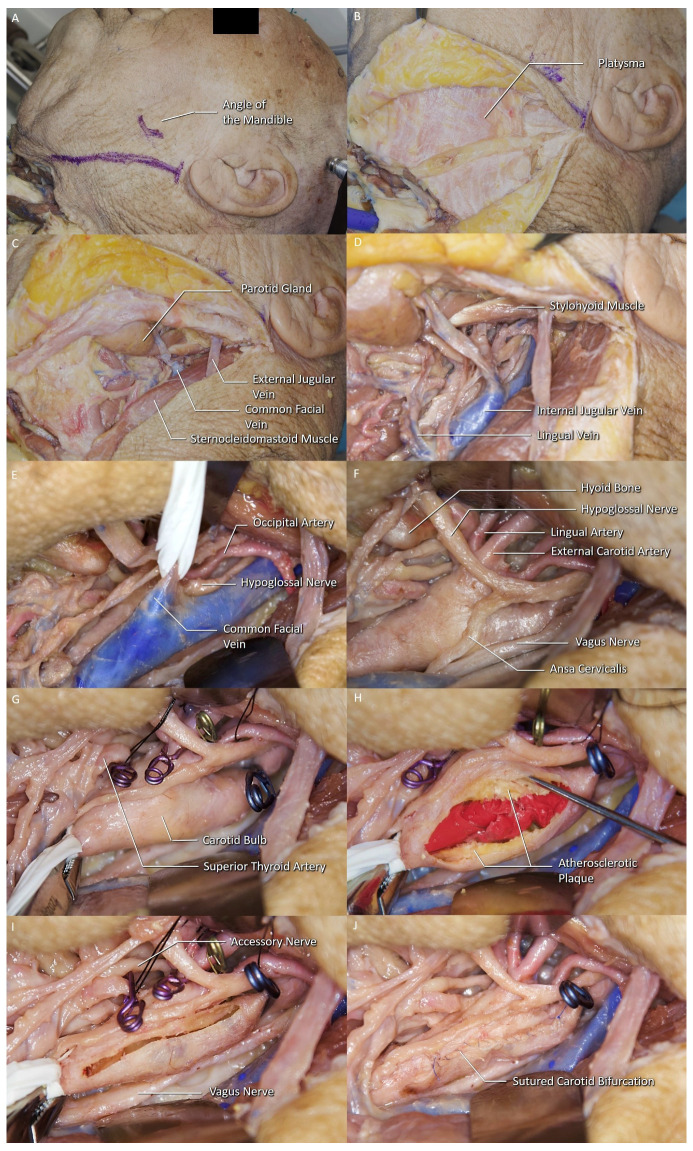
Stratigraphic dissections following the classical CEA procedure. (**A**) Marking for skin incision can be seen along the medial edge of the sternocleidomastoid muscle (SCM). (**B**) The divided platysma can be seen. (**C**) The common facial vein can be seen along the medial edge of the SCM. (**D**) The IJV is exposed with its tributaries, common facial and lingual vein. (**E**) Common facial vein is isolated prior to its ligation. (**F**) The IJV was retracted laterally to expose the hypoglossal nerve (HN) and the carotid bulb (carotid sinus). (**G**) The view of the carotid bulb after crossclamping. (**H**) A longitudinal incision on the carotid starting from the proximal end of the plaque extending to the internal carotid artery (ICA) can be seen. (**I**) The carotid bulb after the plaque removal. (**J**) The sutured carotid and the last clamp on the ICA can be seen.

44 sides of the 22 formalin-fixed cadaveric heads were prepared for anatomical dissection at the Department of Neurosurgery, “Center for Advanced Simulation and Education (CASE), Neuroanatomy Lab” at Acibadem MAA University. Stratigraphic anatomical dissections were carried out, following the classical carotid endarterectomy procedure, and results were photographed at every step of the procedure (Figure 1).

## 3. Results

**Figure 2:**
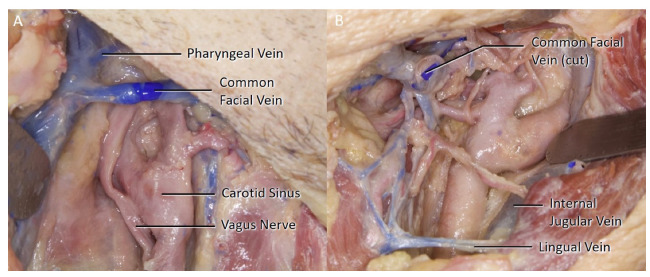
(**A**) A direct right side pharyngeal vein along with a common facial vein can be seen. (**B**) A left side cut common facial vein, a direct lingual vein inferiorly and the venous interconnections medially (left) can be seen.

44 sides of 22 cadaveric heads were dissected following the classical CEA procedure. The female to male ratio of the specimens were 47.6%. In 20 of the 44 sides, there were more than one branches of the internal jugular vein (IJV) identified within the surgical exposure (45.5%) (Table 1). A facial vein was identified in 38 sides (86.4%), at least one pharyngeal vein in 22 sides (50%) (Figure 2A), and one direct lingual vein in four sides (9.1%) (Figure 2B). A single facial vein was the only branch seen in 16 of the sides (36.4%), and a pharyngeal vein in two (4.5%). In four sides (4.5%), no IJV branches were encountered within the surgical area which led to a carotid exposure without any ligation of the veins.

### 3.1. Common Facial Vein

**Figure 3:**
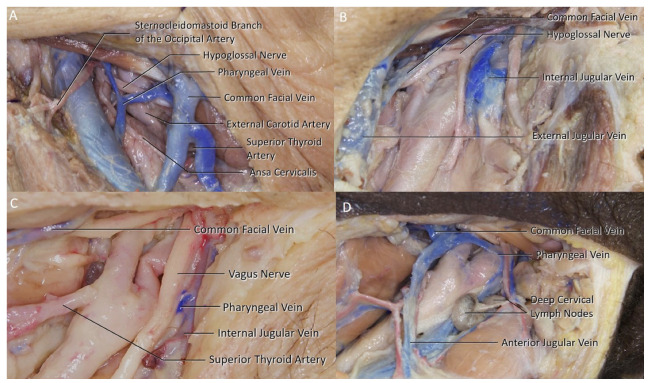
(**A**) Left side pharyngeal vein can be seen draining into the IJV separately from the common facial vein. (**B**) No ligation was required on the common facial vein as it drained into the external jugular vein. (**C**) Left side pharyngeal vein can be visualized as the only branch of the IJV at the surgical site. (**D**) Left side anterior jugular vein drains the common facial vein, in addition, a communicating vein between the common facial and pharyngeal vein can be seen.

The common facial vein is formed by the union of the anterior facial vein and the posterior division of the retromandibular vein leaving the parotid gland. The anterior facial vein is the extent of the angular vein formed by the supratrochlear and the supraorbital veins. The retromandibular vein on the other hand is formed by the superficial temporal veins and the retromandibular vein. The remaining posterior division of the retromandibular vein does not drain into the IJV and continues as the external jugular vein after joining with the posterior auricular vein. The common facial vein was identified in 86.4% of the sides. In 20 sides (45.5%), the common facial had an accompanying branch draining into the IJV (Figure 3A). No common facial vein was identified within the surgical field in six sides (13.6%). The common facial vein was draining into the external jugular vein (EJV) (Figure 3B,C) in two sides. Additionally, a connecting vein was identified between the common facial vein and the anterior jugular vein (Figure 3D) in two sides.

### 3.2. Pharyngeal Vein

**Figure 4:**
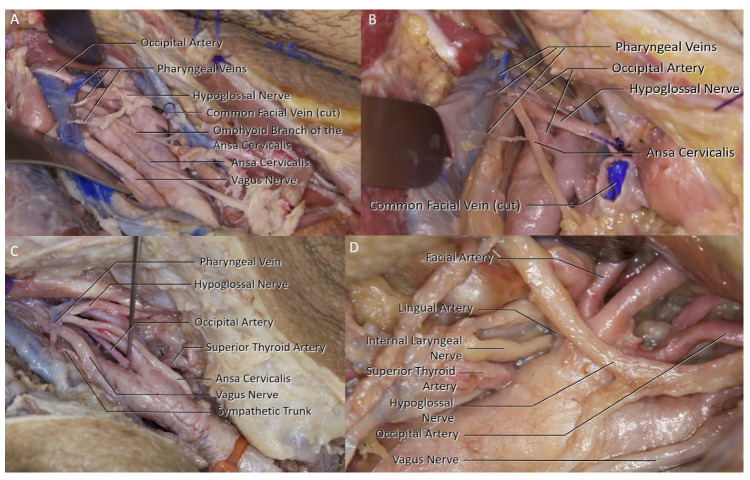
(**A**) Multiple pharyngeal veins can be seen on this right sided approach. (**B**) The right-side occipital artery can be seen pushing the HN away from the carotid bifurcation. (**C**) Right side pharyngeal vein can be seen intersecting the HN. (**D**) Left side HN can be seen neighboring the carotid bulb, an ansa cervicalis division may be required for sufficient mobilization of the HN.

The pharyngeal vein collects the venous blood of the pharynx and drains into the IJV (Figure 3C). A pharyngeal vein was identified in 50% of the sides. In two sides, the pharyngeal vein was the only branch of the IJV at the surgical area (Figure 3C). In two sides, there were multiple pharyngeal veins draining into the IJV (Figure 4A,B).

### 3.3. Lingual Vein

The lingual vein drains into the IJV at the level of the greater cornu of the hyoid bone. Four separate lingual vein branches were identified (18.18%) within the surgical area. The separate lingual vein was accompanied by a common facial vein in two sides, and both common facial and pharyngeal veins in the remaining two sides. The four separate lingual veins had no close relationship with any other structures (Figure 2B).

### 3.4. Arterial Branches

The superior thyroid artery branched as a separate trunk at the level of the carotid bulb (carotid sinus) in 18 sides (40.9%) (Figure 4C,D), followed by the base of the medial aspect of ECA in 12 sides (27.3%) (Figure 4A,B). No other arterial branches were observed coming off the carotid bulb. In one side, the occipital artery was seen pushing the HN superolaterally away from the carotid bulb (Figure 4B).

### 3.5. Carotid Bifurcation

In 28 sides (63.6%), the carotid bifurcation was at the level of the C3 vertebra. In eight sides (18.2%), the carotid bifurcation was at the C2 level (18.2%) and in the remaining eight (18.2%), the bifurcation was at the C4 level.

### 3.6. Hypoglossal Nerve

The HN was observed at levels varying between the anterior belly of the digastric muscle to as low as the carotid bifurcation where it would require mobilization to gain access to the carotid bulb. A HN was identified in all but four sides. In those four sides, the HN was considered to make its horizontal turn more superiorly; therefore, not entering the surgical field of view. 16 of the 44 sides had the HN in proximity of the surgical path (36.7%). Four of the hypoglossal nerves required extensive mobilization possibly with an ansa cervicalis superior branch sacrifice (Figure 4A,D). It can be divided to release the HN without introducing major functional or cosmetic consequences [5]. The superior root of the ansa cervicalis was identified within the surgical area in 28 of the 44 sides (63.6%). In two sides, the omohyoid branch could be observed, leaving the superior branch, inferior to the hypoglossal nerve-ansa cervicalis bifurcation (Figure 4A). Although it has been shown in the literature that variations of the superior branch are rarer than the inferior branch, such variations are possible [6].

### 3.7. Side Variation

Although slight variations in all structures between right and left sides were present in all specimens, 16 of the 22 specimens had considerable variances between the two sides in the venous system (72.7%). The branching patterns within the surgical field between the two sides were reported as single facial, single pharyngeal, pharyngeal and facial, facial and lingual, and finally as all three veins observed. On the right side the most common pattern was pharyngeal and facial, seen in 15 of the specimens (68.2%). On the left side the most common branching was a single common facial as seen in 12 of the specimens (54.5%). The results are reported in Table 1.

## 4. Discussion

Cranial nerve injuries have remained as significant complications since the early days of CEA. According to the most recent meta-analysis, the most injured cranial nerve is the vagus (3.99%), with the hypoglossal following (3.79%) [4]. Majority of these reported cranial nerve injuries are transient, usually recovering within 6 to 12 months. The glossopharyngeal nerve has the highest rate of recovery while the vagus has the lowest. In the CEA procedure, the most common reported mechanism of injury is trauma caused by retraction, dissection, cauterization and clamping of the nerves [3,4]. There are very few studies in the literature that deal with the role of venous anatomy on cranial nerve damage mechanisms in detail. To this date, only one study had used the term ’pharyngeal vein’ in a study regarding cranial nerve injuries in CEA. Aldori et al. discussed the relationship between the presence of the pharyngeal vein in the surgical field with the injury of the HN during CEA [7]. The study have suggested that during the removal of the plaque, this small venous branch may bleed due to retraction of the internal jugular vein. HN injury due to diathermy was found to be surprisingly high at the bleeding control stage [7]. The authors reported that their HN injury rate significantly lowered after the pharyngeal vein branch was actively searched prior to plaque removal. Besides the pharyngeal vein, it has been reported twice by another author that vena comitans hypoglossi also causes cranial nerve damage in similar fashion, therefore proposed to be ligated when found [8,9]. The vena commitans hypoglossi which is the main drainage vein of the HN that usually drains to the facial, lingual, or superior thyroid veins was not observed to be a limiting factor on the exposure in our dissections; therefore, it could be speculated that the author may have been referring to the pharyngeal vein [10]. In the final study mentioning the venous system as cranial nerve injury risk factor, it has been suggested that ’any small vein crossing the carotid bifurcation’ should be ligated [11]. These clinical studies have shown that venous variations and their proximity to the surgical field are linked to an increase in cranial nerve injury risk. To this date, these are all the studies discussing this mechanism. In our study, the venous branching of the IJV at the surgical field was observed and reported. Although it has been shown that many variations can be observed in IJV branching, no studies yet focused on the IJV branching within the CEA surgical field [12]. Apart from the common facial vein seen in 86.36% of the sample, smaller vessels were not few: 50% of the specimens had at least one pharyngeal vein and 9.1% one lingual vein (Table 1). Out of the 22 sides with a pharyngeal vein present, 12 of them were in proximity of the carotid bifurcation (27.3%). None of the lingual veins posed such proximity. It was observed that when the IJV was retracted away from the carotid, these pharyngeal veins were strained, suggestive of a breakage was possible (Figure 4A,B). When such veins are present and not ligated prior, abrupt bleeding from those veins are possible during the plaque removal stage of the surgery where the IJV is retracted farthest away from the carotid bulb. The following bleeding control by diathermy may lead to cranial nerve injuries. The injury risk could naturally be higher if a cranial nerve was present in the vicinity. Considering this possibility, the relation of the pharyngeal vein and the HN was investigated. The two intersected in 40.9% of the sides. 10 of those intersections were located in proximity of the carotid bifurcation (22.8%). Considering the previous clinical studies [4,7], combination of these variations can be considered a risk factor for HN injury. Therefore, it is important for the surgeon to mind these variations as the rate of them being present together is relevant at 22.8%.

Considering the side variations observed in the venous branches as reported in Table 1, the results were further studied. The most common branching pattern seen on the right side was the common facial with at least one pharyngeal vein accompanying, and on the left side was a single common facial. A pharyngeal vein, regardless of branching, was also identified more on the right side with 15 to 7. Therefore, it can be said that, for the area observed on a CEA approach, the pharyngeal vein is seen more common on the right side. The clinically important variations previously discussed with side lateralization data are presented in Table 2. On the right side the pharyngeal vein and HN intersection in proximity of the carotid bifurcation variation was seen in 40.91% of the 22 specimens. For the left side the rate was 4.55%. Although this variation rate was expected to be increased on the right side due to the increased incidence of pharyngeal veins on the right side; the rate difference was higher than anticipated. The p value for the side difference is calculated to be 0.0114 when compared to an even side distribution. Looking at the literature, there are no studies reporting substantial side differences in anatomical variations regarding CEA procedure. One study reported considerable asymmetry between contralateral sides in vagus nerve position compared to the carotid bifurcation [13]. In a study reporting side specific cranial nerve injury data on their series, no major difference between sides were reported for HN [14]. The reason behind this side difference can be linked to the variation between sides in the embryological development of the venous system of the neck. After the early embryological development, the symmetrical anterior cardinal veins go through segmental fusion and regression, shifting more to the right atrium in the process, and form the left brachiocephalic vein [15]. It can be argued that this developmental side difference leads to the side variation differences found in this study. These anatomical variations should be considered by the surgeon in right-sided approaches.

**Figure 5:**
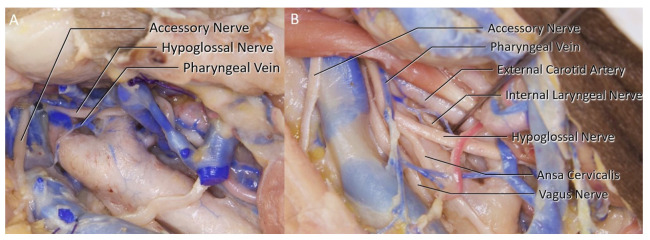
(**A**) Right side carotid bifurcation at the level of C2 vertebra. The hypoglossal nerve can be visualized but a mobilization may not be necessary. (**B**) The right-side hypoglossal and vagus nerve can be visualized as the internal carotid artery bifurcates at the level of the C2 vertebra.

The backbleeding is a complicative event that might be encountered if any of the branches of the carotid system is not clamped. To negate such possibility, it is important have a good knowledge of the common possible variations of the branching patterns. Superior thyroid artery (STA) most often branches off the carotid bulb followed by the anterior aspect of the ECA [16,17]. In our series, the STA was found to branch most commonly from the carotid bulb (40.9%). It must be stressed that the medial aspect of the external carotid system should be dissected thoroughly to visualize if there is any branching at the level of the bulb. Another important feature of the arterial anatomy in the CEA procedure is the relationship between the HN and the carotid bifurcation. It has been shown in the literature that when the HN was observed in proximity of the carotid bifurcation, the risk of a HN injury was higher [4]. It has also been reported previously that a higher level of carotid bifurcation is linked to a higher rate of HN proximity [17]. Accordingly, the dissection data in this study is elaborated further to include carotid bifurcation levels, in a way to correlate the carotid bifurcation levels with HN proximity. Consequently, if varying levels showed different HN proximity rates, an assessment of HN injury risk depending on the carotid bifurcation levels can be made. The carotid bifurcation level and HN proximity observed in the study are reported in Table 3. We have observed carotid bifurcation at C2 vertebra level on eight of the 44 sides (18.2%) which was slightly higher than previously reported [18,19]. Out of these eight sides, four HN were in proximity of the bifurcation (50%) (Figure 5A,B). On 28 sides with C3 level carotid bifurcation, 12 had a HN in proximity (42.9%). In the remaining eight sides with lower carotid bifurcation at the level of C4, HN was not in proximity (0%). It was observed that as the bifurcation level moved superiorly, the HN proximity rate increased, suggestive of a higher bifurcation being related to a higher HN injury risk. In the same study reporting side lateralization data for the HN injury, it has been mentioned that both HN injury cases had a high carotid bifurcation which required the use of a Langenbeck retractor to lift the HN [14]. Hence it should be noted that if a higher bifurcation is predicted from the preoperative imaging, an HN encounter should be expected and the surgeon should be prepared to mobilize the HN to lower the risk of injury. It is also important to point out that all eight sides with carotid bifurcation at the C2 level were of African descent. No previous study in the literature has reported if the African population have an increased rate of higher carotid bifurcation level. Nevertheless, there is one study discussing a higher rate of variation of carotid bifurcation levels in the Kenyan population [20]. This higher incidence of high bifurcation level encountered in this study could be linked to a possible higher variation rate as the study suggests. If a higher carotid bifurcation level was to be linked to African population, that could mean a higher inherent HN injury risk during CEA.

## 5. Conclusions

Cranial nerve injuries remain one of the major contributor to the postoperative morbidity of the CEA procedure. Considering the previous studies in the literature and the dissections performed in this study, anatomical variations within the surgical field are not uncommon. Concurrence of two variations was found to be important in HN injury: the presence of a direct smaller vein in proximity of the carotid bifurcation, and the intersection of the HN with this vein. Awareness of possible anatomical variations and early ligation of any small veins can significantly decrease iatrogenic injury risk.

## Figures and Tables

**Table 1:** Venous Branches.

Attribute	Right	Left	Total
Total Specimens Available	22	22	44
No IJV Branch	2	2	4
Facial Vein	20	18	38
Pharyngeal Vein	15	7	22
Lingual Vein	1	3	4
Single Facial Vein	4	12	16
Single Pharyngeal Vein	0	2	2
Pharyngeal + Facial Veins	15	3	18
Facial + Lingual Veins	1	1	2
All 3 Branches	0	2	2
Multiple Pharyngeal Veins	2	0	2

**Table 2:** Side lateralization results.

	Total Sides	Right	Left	*p* Value
Phayngeal Vein over the Bifurcation	12 (27.3%)	9	3	0.0833
PV and HN Intersection	18 (40.9%)	13	5	0.0593
Both Variations Present	10 (22.8%)	9	1	0.0114

**Table 3:** Carotid Bifurcation level and the rate of having a HN in proximity.

Bifurcation Level	Number of Sides	HN in Close Proximity	Rate
C2	8	4	50%
C3	28	12	42.9%
C4	8	0	0%

## Data Availability

Data is contained within the article.

## Abbreviations

The following abbreviations are used in this manuscript:

CEA	Carotid Endarterectomy
HN	Hypoglossal Nerve
CASE	Center for Advanced Simulation and Education
SCM	Sternocleidomastoid Muscle
IJV	Internal Jugular Vein
CCA	Common Carotid Artery
ICA	Internal Carotid Artery
ECA	External Carotid Artery
STA	Superior Thyroid Artery
